# Energy Efficient Range-Free Localization Algorithm for Wireless Sensor Networks

**DOI:** 10.3390/s19163603

**Published:** 2019-08-19

**Authors:** Rekha Goyat, Mritunjay Kumar Rai, Gulshan Kumar, Rahul Saha, Tai-Hoon Kim

**Affiliations:** 1 School of Electronics and Electrical Engineering, Lovely Professional University, Phagwara, Punjab-144411, India; 2 School of Computer Science and Engineering, Lovely Professional University, Phagwara, Punjab-144411, India; 3 Division of Research and Development, Lovely Professional University, Phagwara, Punjab-144411, India; 4 School of Economics and Management, Beijing Jiaotong University, No.3 Shangyuancun, Beijing 100044, China

**Keywords:** Wireless Sensor Network, localization, accuracy, range-free, DV-Hop algorithm

## Abstract

In this paper, an energy-efficient localization algorithm is proposed for precise localization in wireless sensor networks (WSNs) and the process is accomplished in three steps. Firstly, the beacon nodes discover their one-hop neighbor nodes with additional tone requests and reply packets over the media access control (MAC) layer to avoid collision of packets. Secondly, the discovered one-hop unknown nodes are divided into two sets, i.e. unknown nodes with direct communication, and with indirect communication for energy efficiency. In direct communication, source beacon nodes forward the information directly to the unknown nodes, but a common beacon node is selected for communication which reduces overall energy consumption during transmission in indirect communication. Finally, a correction factor is also introduced, and localized unknown nodes are upgraded into helper nodes for reducing the localization error. To analyze the efficiency and effectiveness of the proposed algorithm, various simulations are conducted and compared with the existing algorithms.

## 1. Introduction

With the rapid development of microelectromechanical systems (MEMS), very-large-scale integration (VLSI) and wireless communication technology have led to the growth of multifunctional small sensor nodes [1,2,3]. The collection of these small sensor nodes deployed in the environment randomly forms a network called a wireless sensor network (WSN). The sensor nodes perceive the physical activities surrounding the area of interest and forward the detected information to the sink node or base station through the wireless medium for further processing. WSNs are extensively applied in various applications such as industrial application, military application, battlefield, automation, smart city, healthcare, automobiles, and environmental monitoring, etc. [4,5,6,7]. A number of applications such as remote area monitoring, disaster monitoring, military applications, and habitat monitoring, etc. require precise locations of sensor nodes from where the sensed information is forwarded for further computation to make the collected information meaningful [8,9]. In these situations, without knowing the location of the event, the information given by sensor nodes is useless. Hence, the process of determining the positions of sensor nodes is called localization [10,11,12]. Therefore, precise localization of sensor nodes becomes a key issue for researchers of WSNs. The deployment of sensor nodes manually is a simple and easy method for localization, but not feasible for remote areas and large scale deployments. Another simple method of localization is to install sensor nodes with global positioning system (GPS) receivers, but that adds size, cost, complexity, and energy consumption [13,14,15]. Further, localization approaches are broadly classified as range-based or range-free. In range-based approaches, the positions of sensor nodes are computed using distance or angle information while range-free approaches compute the locations using connectivity and hop-count information. In these approaches, some sensor nodes are installed with GPS, known as beacon nodes (BN), and remaining sensor nodes, labelled unknown nodes (UN), determine their position with the help of beacon nodes [16,17,18]. Range-based approaches give more precise localization using various techniques, i.e. angle of arrival (AoA) [19,20], time of arrival (ToA) [21,22], received signal strength indication (RSSI) [23], time difference of arrival (TDoA), etc. [24,25], but they require special hardware for operations and are also affected by multipath fading. Channel state information (CSI) localization based on the Cramér–Rao lower bound (CRLB) range-based algorithm has been developed for indoor localization. The effect of shadowing and the multipath effect are addressed using CRLB [26]. Range-free approaches are widely used due to their low cost and simplicity but suffer from poor localization accuracy [27,28,29]. Also, the operable batteries of sensor nodes have limited energy and these batteries must be replaced or recharged once depleted. Therefore, in the current research paper, the localization accuracy with energy efficiency is improved by the proposed algorithm. Contribution of the research paper is highlighted as follows:

- A range-free, energy-efficient, novel Distance Vector-Hop (DV-Hop) localization algorithm is proposed to accomplish precise localization and energy efficiency.
- The neighbor nodes of beacon nodes are discovered using two additional Nearest Neighbor ReQuest Tone (*NNReQT*) and Nearest Neighbor RePly Tone (*NNRePT*) packets over the media access control (MAC) layer to reduce collisions during transmission.
- Further, one-hop neighbor nodes are distributed in two parts to reduce energy consumption, for instance: nodes with direct communication, and with indirect communication.
- The beacon nodes are selected as common nodes for indirect communication between unknown nodes and beacon nodes to reduced energy consumption.
- Finally, the localization errors are reduced using a correction factor and localized unknown nodes are upgraded into helper nodes for accurate localization.

## 2. Present State of Research and Research Gaps

In the literature, various range-free algorithms have been suggested for precise localization such as centroid [30,31], approximate point in triangle (APIT) [30,32], gradient [33], DV-Hop [34], and multidimensional scaling (MDS) [35], etc. The DV-Hop algorithm has attracted relatively more attention from researchers due to its easiness and low-cost hardware [36,37,38]. However, the DV-Hop algorithm does provide more localization errors during localization, due to hop-count distances. In this section, some variants of DV-Hop are discussed which significantly improve the localization accuracy. A weighted DV-Hop algorithm has been introduced in which the weights of beacon nodes are computed to reduce the localization error [39,40]. Another improved DV-hop localization algorithm is shown in [41,42] where the positions of nodes are computed using the quasi-Newton iterative algorithm. Another improvement of DV-Hop has been shown [43], in which position computation is done by weighted hop-size of ordinary nodes to improve the accuracy. A variant of the DV-Hop algorithm has been introduced where transmission of information packets is limited to k-hops instead of the whole network, in order to reduce the energy consumption during localization in WSNs [44].

It is also observed from the literature that a variant of DV-Hop has utilized the evolutionary optimization algorithm to improve the localization accuracy shown in [45,46,47]. A shuffled frog leaping algorithm (SFLA) with DV-Hop is introduced by [48], in which the modified hop-size is achieved with SFLA. Improved DV-Hop localization algorithms with particle swarm optimization-differential evolution (PSO-DE) and cuckoo search optimization are shown in [49,50], which reduce localization error. A variant of the DV-Hop algorithm has been shown by [51] in which grey-wolf optimization (GWO) is utilized for computing the average hop-size of beacon nodes and a weighted GWO is applied for accurate localization in WSNs. An advanced DV-hop with teaching learning based optimization (TLBO) has been developed by introducing the correction factor and collinearity concept in localization [52,53,54]. Another modified DV-Hop algorithm with differential evolution (DE) has been developed for accurate localization in WSNs. The discrete values of hop-count are transformed into continuous values to reduced localization error [55]. A swarm intelligence based localization algorithm has been introduced for mobility assisted nodes in WSNs. The movements of nodes are controlled by using the grey wolf optimizer (GWO) and whale optimization algorithm (WOA) in a real-time scenarios [56]. The Lloyd-α and distributed energy-efficient self-deployment (DEED) algorithm have been proposed for mobile WSNs. The former algorithm controls the movement step size of nodes, and the latter one limits the traveling distances of the nodes [57]. An advanced hop-count based algorithm is shown in [58] to decrease the localization errors in WSNs. A weighted Monte Carlo localization (WMCL) based algorithm has been proposed in mobile WSNs to reduce energy consumption. The size of the bounding-box for nodes is reduced to upgrade the sampling efficiency and accuracy of localization [59]. Another variant of the DV-Hop algorithm has been developed based on the non-dominating sorting genetic algorithm-II (NSGA-II) for the internet of things (IoT) [60]. 

Recently, some research on cooperative MAC protocols have been developed [61,62]. The MAC protocols utilized for WSNs with their performance is shown in [63]. Cooperative communication at MAC has been shown in [64] where optimization problems have been formulated for one-hop as well as multi-hop communication. A complete overview of the evaluation of MAC layer protocols has been introduced in [65] for underwater WSNs. A cooperative MAC has been considered for underwater WSNs in which transmission energy optimization techniques are incorporated in order to prolong the network lifetime [66]. A cross-layer distributed cooperative MAC has been identified where the best cooperative nodes are selected on the basis of residual energy and location information [67]. A new medium access control (MAC) for the DV-HOP localization algorithm has been proposed using the Chinese remainder theorem (CRT) protocol sequence to improve the packet transmission success rate [68].

From the literature, it can be observed that all the variants of DV-hop algorithms have tried to minimize the localization error to some extent, but the energy-conserving concept over the MAC layer has been less considered by the researchers. The network lifetime of WSNs can be prolonged by either maximizing the energy of the nodes or minimizing the energy consumption in the network. Also, strict timing is required during communication between sensor nodes to avoid collisions, and the strongest impact for indirect communication is provided over the MAC layer. However, the localization accuracy and energy efficiency are still challenging issues for the localization process. By considering both facts, we proposed an energy-efficient novel DV-Hop algorithm for better localization with the least energy consumption.

The rest of the paper is explained as follows. The proposed algorithm is discussed in Section 3. The performance evaluation of the present work is presented in Section 4 followed by the conclusion of the study in Section 5.

## 3. Proposed Network Model

In the network model, total *N* sensor nodes are organized randomly in a two-dimensional area without any central control. The sensor networks are represented by G(*V*, *E*) in which *V* defines the set of vertices and *E* describes the set of edges. Total *m* sensor nodes are deployed, whose locations are predefined in advance using GPS receivers known as beacon nodes. The remaining *n* sensor nodes are recognized by unknown nodes whose locations are to be estimated using beacon nodes. The unknown nodes whose locations are discovered in the first iteration will act as helper nodes and provide assistance to all beacon nodes for finding the locations of remaining unknown nodes. The size of the network model is defined as:

|N|=|m+n|.

### 3.1. Anisotropic Network Model

In real-time network scenarios, the sensor nodes do not have a perfectly circular area of communication or radiation pattern. All the sensor nodes are independently and randomly deployed in a two-dimensional sensing area with their unique identification (Id). The radiation pattern is affected by various parameters such as fading, path losses, interference, and shadowing effect causing irregularities in the radiation pattern. These irregularities are a critical issue of concern and cannot be ignored in wireless communication. Consequently, the impact of irregularity is also explored in the proposed approach and the radio irregularity model [69] is considered for the same. A parameter degree of irregularity (DOI) is introduced to determine the irregularity of the radio pattern and it is defined as the approximate change in radio range per unit degree variation in direction.

**Figure 1:**
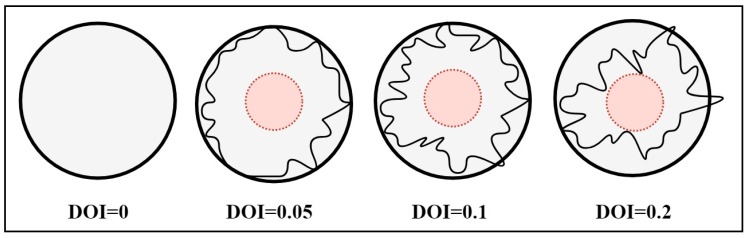
Radio pattern with degree of irregularity (DOI).

Irregularity of radio pattern in the Radio Irregularity Model (RIM) model with different DOI values is depicted in Figure 1.

### 3.2. Proposed Algorithm

**Figure 2:**
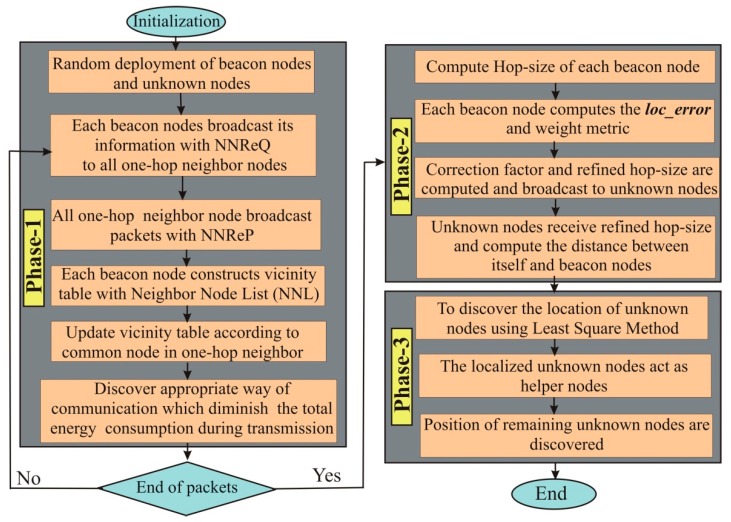
Flowchart of the proposed methodology. Nearest Neighbor Request (NNReQ) and Nearest Neighbor Reply (NNReP).

In this section, we proposed an energy-efficient novel DV-Hop localization algorithm. A traditional DV-Hop algorithm is completed in three phases: Firstly, the beacon nodes forward their location to the one-hop neighbor sensor nodes; secondly, hop-size distance of each beacon node is computed; and the positions of unknown nodes are discovered in the last phase. The proposed algorithm is also completed in three phases as depicted in Figure 2. Detailed description of the proposed algorithm is as following:

• ***First Phase***

**Figure 3:**
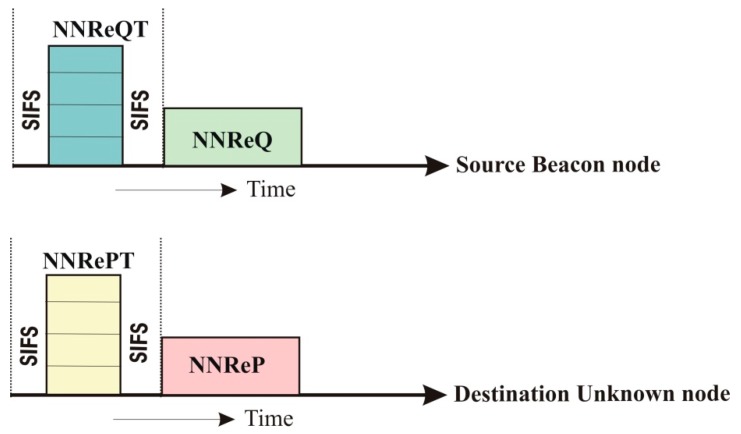
Neighbored node discovery. Nearest Neighbor Request Tone (NNReQT) and Nearest Neighbor Reply Tone (NNRePT), short inter-frame space (SIFS).

In the first phase of the proposed algorithm, beacon nodes discover their one-hop neighbor nodes by forwarding the *Nearest Neighbor Request* (*NNReQ*) and *Nearest Neighbor Reply* (*NNReP*) packets same as 802.11. Additional two packets are also included in the proposed algorithm to avoid collision of packets during transmission. These two packets are *Nearest Neighbor Request Tone* (*NNReQT*) and *Nearest Neighbor Reply Tone* (*NNRePT*) shown in Figure 3 and are forwarded before broadcasting *NNReQ* and *NNReP*. Initially, the *NNReQT* is broadcasted by beacon nodes called source beacons ($Source_{BN}$) bounded with short inter-frame space (SIFS) which is used between each frame to ensure that no other radios are transmitting during the transmission. Information packets are forwarded for finding the neighbor unknown nodes called the destination unknown $\left(Destination_{UN}\right)$ nodes after the SIFS time has elapsed. Next, the *NNReQ* packet is broadcasted if the *NNReQT* is received successfully by neighbor nodes. Now, $Destination_{UN}$ transmits the *NNRePT* followed by *NNReP* packets to $Source_{BN}$. The format of *NNReQ* and *NNReP* are as follows:

$$NNReQ\to Destination_{UN}:\text{\{}Id_{i},\left(x_{i},\;y_{i}\right),\;E_{residual},\;E_{direct},\;E_{indirect},\;({\mathrm{Hop}}_{\mathrm{count}}=0)\}$$

$$NNReP\to Source_{BN}:\text{\{}Id_{i},\left(x_{i},\;y_{i}\right),\;Id_{u},\;({\mathrm{Hop}}_{\mathrm{count}}=1)\}$$

where $Id_{i}$ and $Id_{u}$ represent the identification of the beacon node and unknown node respectively. $\left(x_{i},\;y_{i}\right)$ is the position of beacon node, $E_{residual}$ represents the residual energy and $E_{direct}\;\mathrm{and}\;E_{indirect}$ are the energy required during direct communication and indirect communication respectively. 

In this way, each beacon node enlists its one-hop neighbor unknown nodes in a neighbor node list (NNL) within its transmission range. Let $E_{max}$ be the maximum energy, $E_{min}$ represent the minimum energy, and $E_{T}\left(b\right)$ be the required transmission energy of the beacon node (b∈m). The transmission energy ($E_{T}$) can be adjusted within the range of maximum and minimum energy, $E_{min}\leq E_{T}\leq E_{max}$. Let $E_{b,u}$ be the minimum energy required for data transmission between the b and u nodes and it is computed as follows:

(1) $$E_{b,u}=Distance_{\left(b,u\right)}^{\alpha }+C$$

where α represents the path loss exponent, $Distance_{b,u}$ denotes the Euclidean distance among beacon node *b* and unknown node *u* and *C* are the constants. The energy model for the proposed algorithm is the same as [70] and the cost of k-bit packet transmission to a distance $Distance_{\left(b,u\right)}$ is shown in Equation (2):

(2) $$E_{T}^{j}\left(k,Distance_{\left(b,u\right)}\right)=kE_{elec}+kE_{amp}{\left(Distance_{\left(b,u\right)}\right)}^{2}$$

where $E_{T}^{j}$ is the energy required for transmission and $E_{elec}$ and $E_{amp}$ are the required energy for electronic circuitry and amplification respectively in the aforementioned Equation.

(3) $$E_{R}^{j}\left(k\right)=kE_{elec},\;\;\;\;\;\;\left(j=1,2,3,\ldots .,m\right).$$

$E_{R}^{i}\left(k\right)$ represents the required energy to receive k-bit information within the transmission range. Hence, the total energy consumption of the beacon node is computed by the following equation:

(4) $$E_{consumption}^{j}=E_{T}^{i}\left(k,Distance_{\left(b,u\right)}\right)+E_{R}^{i}\left(k\right).$$

Now the residual energy ($E_{residual}^{j}$) is computed as follows:

(5) $$E_{residual}^{j}=E_{Initial}^{j}-E_{consumption}^{j}.$$

**Figure 4:**
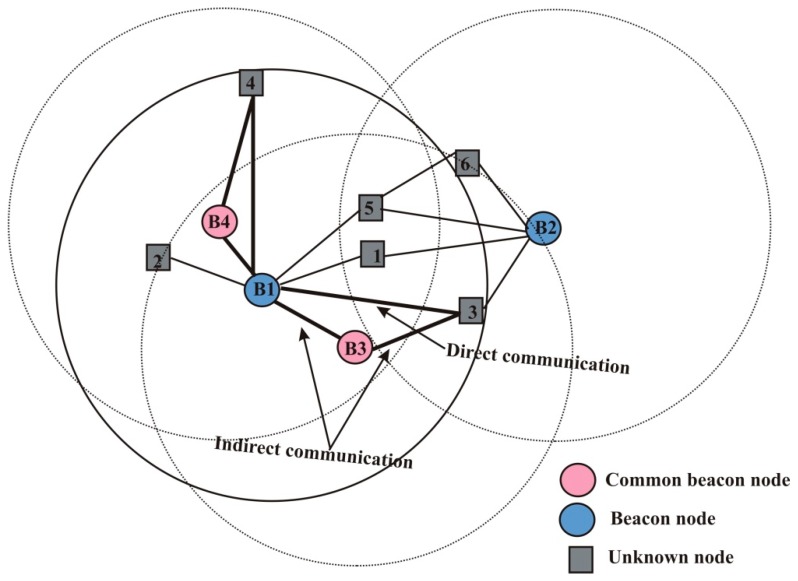
Unknown nodes with direct and indirect communication.

The set of unknown nodes present in the NNL are also divided into two groups for energy-efficient transmission: nodes with direct communication, and with indirect communication. Unknown nodes in the network may be located at a far distance from beacon nodes within one-hop as shown in Figure 4, where unknown node 3 is situated at the border far from B1 and direct communication would consume more energy for transmission. Therefore to reduce energy consumption all sensor nodes communicate together in a peer to peer manner called indirect communication, and unknown nodes select B3 as a common node for indirect communication.

***Neighbor Set:*** Each beacon node enlists its neighbor beacon nodes within one-hop and these nodes can act as a common node during transmission. The common beacon node between the unknown node and beacon node is indicated by $Com^{BN}$ and selected only when it is capable of reducing the overall energy consumption as compared to direct communication. The unknown nodes which have direct communication with beacon node b are denoted with $N_{u}^{direct}$ and with indirect communication are denoted by $N_{u}^{Indirect}$.

**Figure 5:**
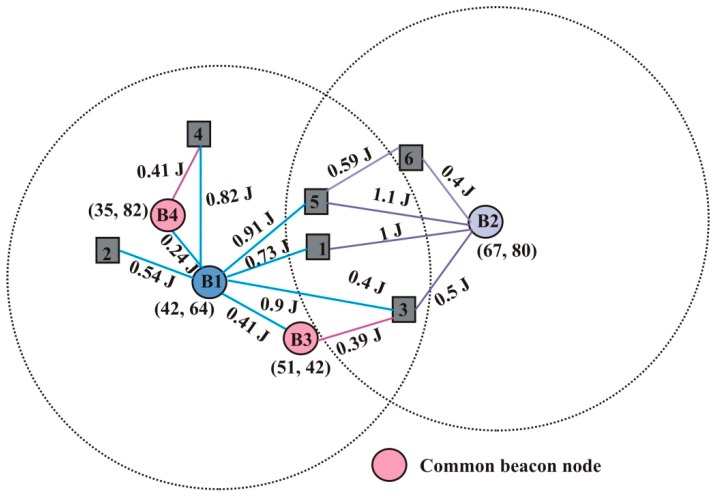
Node distribution with communication cost.

***Neighbor node selection*:** In this phase, the beacon nodes differentiate the one-hop neighbor unknown nodes with direct and indirect communication. As depicted in Figure 5, B1, B2, B3, and B4 are beacon nodes and remaining are the unknown nodes. Let the B1 beacon node broadcast NNReQ to node 1. Node 1 forwards NNReP to beacon node B1. B1 stores the neighbor information in Table 1. Node Id in Table 1 represents the identification or a serial number of sensor nodes and the positions of neighbor beacon nodes are stored in the second column of Table 1. Indirect transmission cost is considered when the $Source_{BN}$ communicates to other $Destination_{UN}$ through intermediate or $Com^{BN}$. During communication between B1 and node 3, B3 act as $Com^{BN}$ and the indirect transmission cost is computed as follows:

(6) $$\mathrm{Indirect}\;\mathrm{transmission}\;\cos t=E_{consumption}^{SC}+E_{consumption}^{CD}$$

where $E_{consumption}^{SC}$ is the energy consumption during transmission from $Source_{BN}$ to $Com^{BN}$ and $E_{consumption}^{CD}$ is the energy consumption during transmission from $Com^{BN}$ to $Destination_{UN}$. The energy consumption of nodes is computed using Equation (4). The $Com^{BN}$ is selected by $Source_{BN}$ only when the indirect transmission cost is less than direct cost (indirect transmission cost<direct transmission cost). Information can be transmitted by using any mode of transmission if the cost of both transmissions for direct as well as indirect is the same.

The identification of the $Com^{BN}$ is represented by a common node Id in Table 1. Direct transmission cost between $Source_{BN}$ to $Destination_{UN}$ is represented by the fifth column of Table 1 and is computed directly using as following:

(7) $$\mathrm{Direct}\;\mathrm{transmission}\;\cos t=E_{consumption}^{SD}$$

where $E_{consumption}^{SD}$ is the energy consumption during direct transmission from $Source_{BN}$ to $Destination_{UN.}$ Status describes the mode of transmission for either direct or indirect transmission. In Table 1, if nodes have a direct connection with a beacon node that means status is 0, otherwise it will be 1. In the same way, each beacon node maintains the vicinity table in which all the information of neighbor nodes is stored as shown in Table 2.

After collecting the information, B1 verifies whether any $Com^{BN}$ exists between itself and its neighbor unknown nodes. Beacon nodes B3 and B4 are $Com^{BN}$ for nodes 3 and 4 respectively and it may be possible when more than one $Com^{BN}$ is presented within transmission range, but only that $Com^{BN}$ is selected which reduces the transmission energy significantly. Now, B1 updates its vicinity table according to $Com^{BN}$ as shown in Table 3.

From the updated Table 3, it is observed that the direct energy consumption from B1 to 3 is 0.9 J, but its indirect energy consumption is 0.8 J. Similarly, the direct energy consumption of B1 to 4 is 0.82 J whereas indirect is 0.75 J. In a similar manner, all beacon nodes discover their one-hop neighbor nodes.

• ***Second Phase***

In the second phase, the hop-size distance (h_size) of each beacon node is computed using Equation (8):

(8) $$h\_size_{i}=\frac{\sum_{i\neq j}^{m}\sqrt{{\left(x_{i}-x_{j}\right)}^{2}+{\left(y_{i}-y_{j}\right)}^{2}}}{\sum_{i\neq j}^{m}hop\_count_{ij}}$$

where $(x_{i},$ $y_{i}$) and $(x_{j},$ $y_{j}$) are the coordinates of beacon node *i* and beacon node *j* and $hop\_count_{ij}$ represents the number of hop counts between the i and j beacon nodes. The main factor which affects the accuracy is the localization error and it is represented by loc_error. The actual distance between beacon node j and beacon node i is calculated as follows:

(9) $$D_{ij}^{act}=\sqrt{{\left(x_{i}-x_{j}\right)}^{2}+{\left(y_{i}-y_{j}\right)}^{2}}$$

(10) $$D_{ij}^{est}=h\_size_{i}\times hop\_count_{ij}$$

(11) $$loc\_error_{i}=D_{ij}^{act}-D_{ij}^{est}.$$

For precise localization of nodes, the factor loc_error should be minimized as much as possible. A correction factor is introduced to reduce the loc_error as follows:

(12) $$\alpha_{i}=\left\{\frac{\left|D_{ij}^{act}-D_{ij}^{est}\right|}{\left|D_{ij}^{act}+D_{ij}^{est}\right|}\right\}$$

(13) $$w_{i}=\frac{1}{\sum_{i\neq j}^{m}hop\_count_{ij}}$$

(14) $$\rho_{i}=rand\left(\frac{1}{\alpha_{i}+w_{i}}\right).$$

Now the average of the correction factor for all beacon nodes is computed as follows:

(15) $$\varphi =\frac{\sum_{i}^{m}\rho_{i}}{m}.$$

Now the hop-size distance for each beacon node is modified as follows:

(16) $$h\_size_{i}^{modified}=(h\_size_{i}+\varphi ).$$

Each beacon node broadcasts $h\_size_{i}$ to their respective unknown nodes in the network. Each unknown node computes the distance between beacon nodes and itself by using the following:

(17) $$D_{iu}=h\_size_{i}^{modified}\times hop\_count_{iu}.$$

• ***Third Phase:***

Finally, the positions of all unknown nodes are computed using the least squares method, in the same manner as [34]. At the last phase, the positions of the unknown nodes are computed with the help of the trilateration method. Let (*x_u_*, *y_u_*) and (*x_i_*, *y_i_*) be the coordinates of unknown nodes and beacon nodes where [*i* = 1, 2, …, *m*] respectively.

(18) $$\{\begin{array}{c} {\left(x_{u}-x_{1}\right)}^{2}+{\left(y_{u}-y_{1}\right)}^{2}=d_{u1}^{2}\\ {\left(x_{u}-x_{2}\right)}^{2}+{\left(y_{u}-y_{2}\right)}^{2}=d_{u2}^{2}\\ :\\ :\\ {\left(x_{u}-x_{i}\right)}^{2}+{\left(y_{u}-y_{i}\right)}^{2}=d_{ui}^{2} \end{array}.$$

The above equation can be written as follows:

(19) $$\left\{ \begin{array}{c} 2\left(x_{i}-x_{1}\right)x_{u}+2\left(y_{i}-y_{1}\right)y_{u}=d_{u1}^{2}-d_{ui}^{2}-x_{1}^{2}+x_{i}^{2}-y_{1}^{2}+y_{i}^{2}\\ 2\left(x_{i}-x_{2}\right)x_{u}+2\left(y_{i}-y_{2}\right)y_{u}=d_{u2}^{2}-d_{ui}^{2}-x_{2}^{2}+x_{i}^{2}-y_{2}^{2}+y_{i}^{2}\\ :\\ :\\ 2\left(x_{i-1}-x_{i}\right)x_{u}+2\left(y_{i-1}-y_{i}\right)y_{u}=d_{u(i-1)}^{2}-d_{ui}^{2}-x_{i-1}^{2}+x_{i}^{2}-y_{i-1}^{2}+y_{i}^{2} \end{array}.\right.$$

The above equation can be represented in a matrix form AX = B

(20) $$A=-2*\left[\begin{array}{c} x_{1}-x_{i}\;\;\;\;\;y_{1}-y_{i}\\ x_{2}-x_{i}\;\;\;\;\;\;y_{2}-y_{i}\\ :\\ :\\ \;\;\;\;\;\;x_{i-1}-x_{i}\;\;\;\;y_{i-1}-y_{i} \end{array}\right]$$

(21) $$=\left[\begin{array}{c} d_{u1}^{2}-d_{ui}^{2}+x_{1}^{2}-x_{i}^{2}+y_{1}^{2}-y_{i}^{2}\\ d_{u2}^{2}-d_{ui}^{2}+x_{2}^{2}-x_{i}^{2}+y_{2}^{2}-y_{i}^{2}\\ \vdots \\ \vdots \\ d_{u\left(i-1\right)}^{2}-d_{ui}^{2}+x_{i-1}^{2}-x_{i}^{2}+y_{i-1}^{2}-y_{i}^{2} \end{array}\right]$$

(22) $$X=\left[\begin{array}{c} x_{u}\\ y_{u} \end{array}\right].$$

The above equation is solved by using the least square method and the positions of unknown nodes are computed. The equation is expressed as follows:

(23) $$X={\left(A^{T}A\right)}^{-1}A^{T}B.$$

The unknown nodes whose locations are estimated in the first iterations of localization transformed into helper nodes. Now, the positions of the remaining nodes are computed by using beacon nodes as well as the helper node. The overall complexity of the proposed solution is *O*(*n*^2^).

## 4. Performance Evaluation

**Figure 6:**
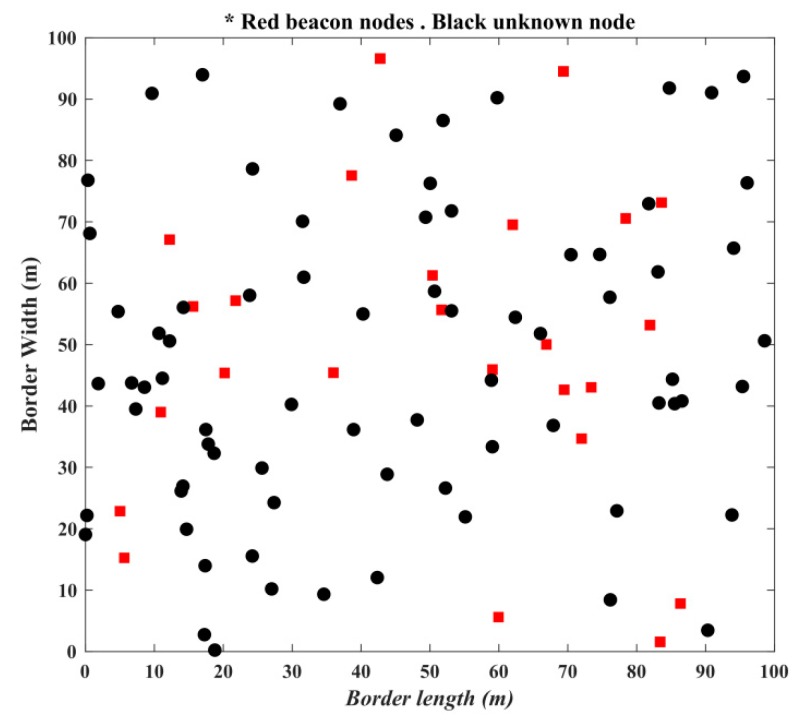
Deployment of sensor nodes.

The simulated results of the proposed algorithm are shown in this section. The simulated results are evaluated in MATLAB with Intel (R) Core(TM) i3-3217 CPU @1.80 GHz. The network scenario is considered static in nature and 100 sensor nodes are randomly deployed with 25% beacon nodes and remaining unknown nodes, as presented in Figure 6. The parameters used in the simulation are presented in Table 4.

To evaluate the performance of the proposed algorithm, various performance metrics are considered as follows:

1. ***Localization error (LE):*** LE error is defined as the difference between the estimated and actual position of unknown node *u* and it is computed as follows:
  (24) $$\mathrm{LE}=\sqrt{{\left(x_{u}^{est}-x_{u}^{act}\right)}^{2}+{\left(y_{u}^{est}-y_{u}^{act}\right)}^{2}}.$$
2. ***Average localization error (ALE):*** ALE is defined as the ratio of the sum of localization error to the total number of unknown nodes and it is computed as follows:
  (25) $$ALE=\frac{\sum_{i=1}^{N-m}\sqrt{{\left(x_{est}-x_{true}\right)}^{2}+{\left(y_{est}-y_{true}\right)}^{2}}}{\left(N-m\right)*R}.$$
3. ***The proportion of placed sensor nodes (PPSN):*** PPSN is defined as the ratio of number of placed sensor nodes $\left(P_{SN}\right)$ to the total number of unknown nodes. When localization error of any particular node is less than (R4), that is called placed node otherwise unplaced node described as following:
  (26) $$UN_{u}=\{\begin{array}{c} \;PPSN\;\;\;\;\;\;\;\;\;\;if\;LE_{u}<\frac{R}{4}\\ PUSN\;\;\;\;\;\;\;\;\;\;\;otherwise \end{array}$$
  (27) $$PPSN=\frac{P_{SN}}{\left(N-m\right)}.$$
4. ***The proportion of unplaced sensor nodes (PUSN):*** PUSN is the ratio of number of unplaced sensor nodes ($U_{SN}$) to a total number of placed nodes and unplaced nodes are those nodes whose locations are not discovered after the localization process. PUSN is expressed as follows:
  (28) $$PUSN=\frac{U_{SN}}{P_{SN}}.$$
5. ***Transmission range:*** In the proposed algorithm, the transmission range of each algorithm is considered variable and varies from minimum to maximum range. The transmission range ($T_{range}$) is computed as follows:
  (29) $$T_{range}=\left(T_{max}-1\right)+Random\left(0,1\right)\times \left[\left(T_{min}-1\right)-\left(T_{max}-1\right)\right]+1$$
  where $T_{min}$ and $T_{max}$ represents the minimum and maximum range of transmission respectively. The transmission range of each beacon node lies in a minimum to a maximum range of transmission.

### Simulated Results

In this section, the simulated results of the proposed algorithm compared with other existing algorithms are described. The simulated results of the proposed algorithm are compared with basic DV-Hop algorithm [34], evolutionary DV-Hop (EDV-Hop) [45], improved DV-Hop (IDV-Hop) [46], and advanced DV-Hop with TLBO (ADV-Hop TLBO) [53]. For evaluation of results, different parameters such as beacon node ratio, node density, sensing field, network connectivity, and simulation time are considered. 

• *Localization error of unknown nodes:*

A total 100 sensor nodes having variable transmission ranges (25–30) are organized with 25% beacon nodes in the simulation area of 100 × 100 m^2^. The performance of each algorithm in terms of maximum, minimum and average localization error is tabulated in Table 5. From Table 5, it is perceived that the proposed algorithm performs better as compared to the existing algorithm.

• *Effect of ratio of beacon node on ALE*

**Figure 7:**
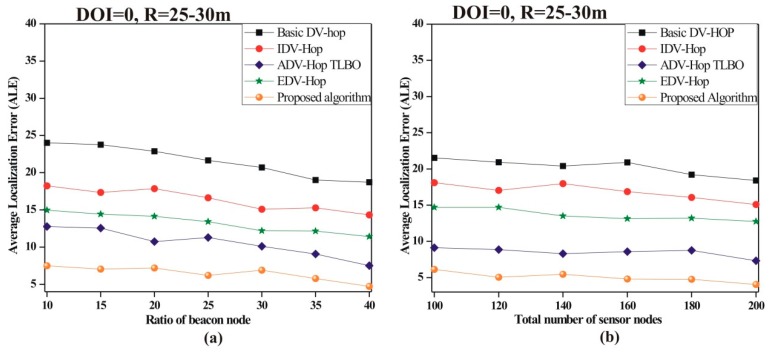
Impact on ALE by varying (**a**) ratio of beacon nodes; (**b**) node density.

To evaluate the performance, the impact of the ratio of beacon nodes on ALE is observed. For this simulation, a total 100 sensor nodes are deployed with 10% to 40% beacon nodes in a simulation area of 100 × 100 m^2^. Figure 7a demonstrates the ALE with respect to the ratio of beacon nodes and it is noticed from simulated results that as the ratio of beacon nodes increases, ALE for each algorithm decreases. It happens because the value of the hop-count decreases with increasing density of beacon nodes.

• *Node density impact on ALE*

The impact of node density on ALE is illustrated in Figure 7b. For this simulation, a total of 100 to 200 sensor nodes are deployed with 25% beacon nodes. Figure 7b represents that, as the density of nodes increases, ALE for each algorithm decreases. The reason for that is as the number of sensor nodes increases, the connectivity between nodes also increases and more location information can be collected from a dense network. From the simulated results, it is noticed that the proposed algorithm identified more precise locations of nodes as compared to existing algorithms.

• *Probability of true location*

**Figure 8:**
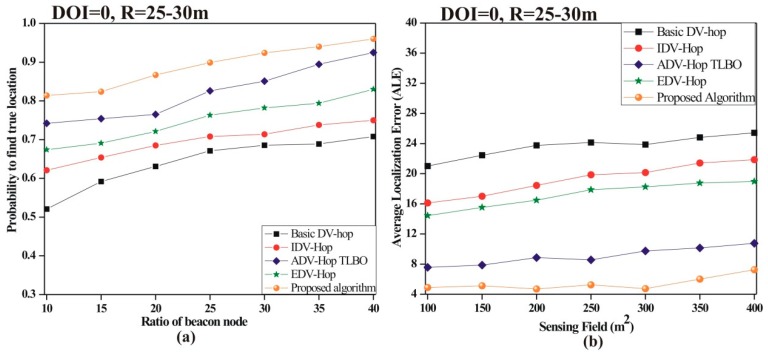
(**a**) Probability of finding the true location; (**b**) impact of the sensing field on ALE.

To analyze the impression of beacon node ratio on the probability of true location, a total of 100 sensor nodes are deployed with 10% to 40% beacon nodes. Figure 8a demonstrates that the probability of finding the true location increases as the beacon node ratio increases for all algorithms. The proposed algorithm has more probability of finding the true location as compared to other existing algorithms.

• *Effect of sensing field on ALE*

To analyze the impact of the sensing area of ALE, a total of 100 sensor nodes are deployed with 25% beacon nodes in the sensing area of 100 × 100 m^2^ to 400 × 400 m^2^. Figure 8b illustrates that the ALE increases as the sensing area increases. This happens due to the fact that as the sensing field increases, connectivity among sensor nodes decreases. From the simulated results it is analyzed that the proposed algorithm accomplishes more accurate localization as compared to the existing one.

• *Effect of network connectivity on localization*

To analyze the influence of network connectivity on localization, PPSN and PUSN is computed by varying network connectivity from 2 to 15. For the simulation, 100 sensor nodes are deployed in the 100 × 100 m^2^ sensing area.

**Figure 9:**
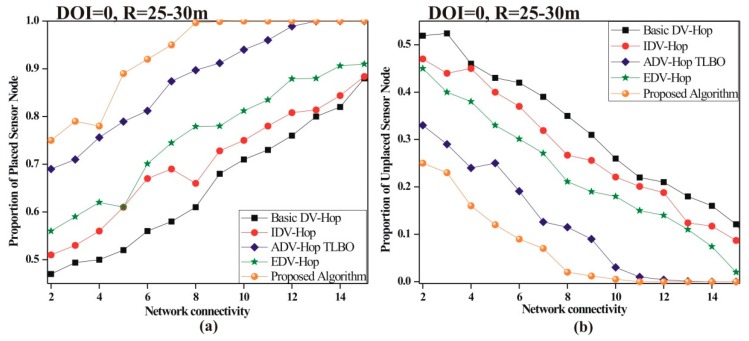
Impact of network connectivity on (**a**) proportion of unplaced sensor nodes (PUSN) (**b**) proportion of placed sensor nodes (PPSN).

The impact of connectivity on PUSN and PPSN is illustrated in Figure 9. Figure 9a demonstrates that as the network connectivity approaches 10, PUSN reaches 0 which specifies that all unknown nodes are effectively positioned. Figure 9b indicates that when network connectivity reaches 10, the PPSN reaches 1, indicating that no unlocalized nodes are present in the process.

• *Impact of radio irregularity on ALE*

**Figure 10:**
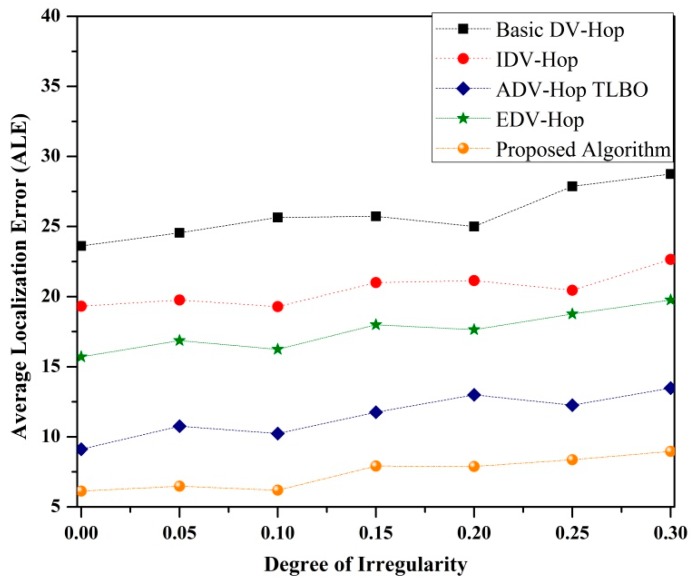
Impact of DOI on ALE.

The irregularity in the radiation pattern of sensor nodes is an important parameter that affects the performance of the localization algorithms significantly. To examine the impact of DOI on ALE, the simulation is conducted by deploying 100 sensor nodes with 25% beacon nodes. Figure 10 demonstrates the impact of DOI on ALE for different algorithms. It is observed from Figure 10 that the ALE of each algorithm decreases as the value of DOI increases. This happens because the connectivity of the network decreases with increment of DOI value, therefore the unknown nodes are unable to collect additional information about their neighbor beacon nodes. From the simulated results it can be seen that the proposed algorithm performs better as compared to other existing algorithms.

• *Impact of transmission range on ALE*

**Figure 11:**
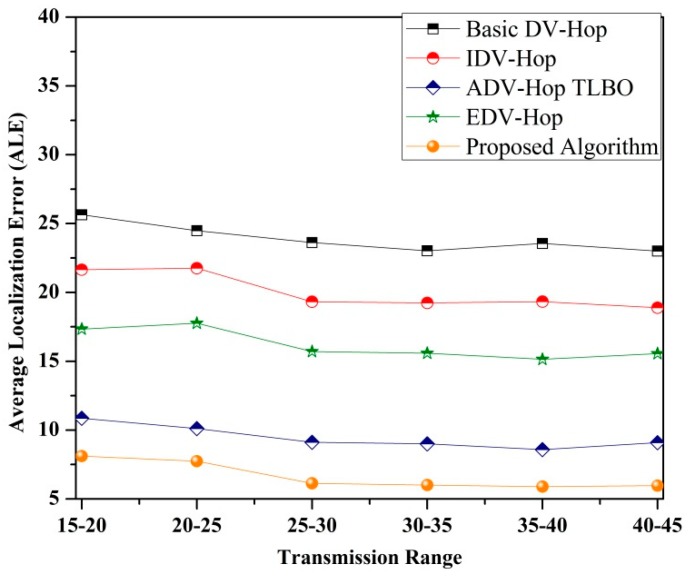
Impact of variable transmission range on ALE (DOI = 0).

Transmission range of sensor nodes also affects the performance of each localization algorithm. To examine the impact of transmission range on ALE, 100 sensor nodes are deployed in a 100 × 100 m^2^ area of sensing with 25% beacon nodes and the transmission range is considered as 15–45 m. Figure 11 demonstrates ALE for different localization algorithm with a varying transmission range of sensor nodes. From the simulated results it is observed that as the transmission range of sensor nodes increases, ALE for each algorithm decreases significantly. Figure 11 also reveals that only minor changes take place in localization errors beyond a 25–30 m transmission range. However, the proposed algorithm accomplishes better localization accuracy as compared to the existing one.

• *Impact of simulation time on residual energy*

**Figure 12:**
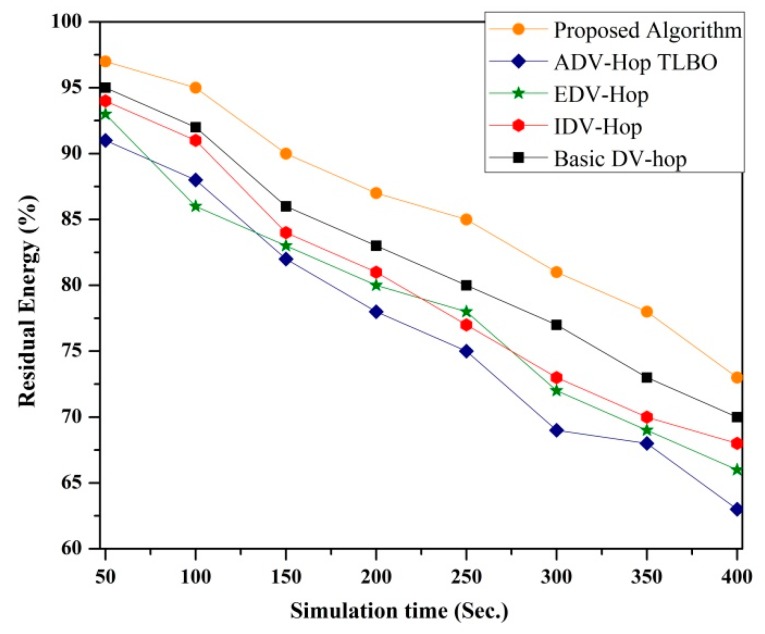
Impact of simulation time on residual energy.

The impact of simulation time on residual energy is illustrated in Figure 12 and a simulation is conducted to compare the percentage of residual energy of the proposed algorithm to other existing algorithms. For the simulation, 100 sensor nodes with 25% beacon nodes are deployed in the 100 × 100 m^2^ sensing area. As the simulation time increases, the percentage of residual energy for each algorithm decreases. From the simulated results it can be observed that the proposed algorithm consumes a small amount of energy to perform localization as compared to the existing one.

Performance of different localization algorithms is summarized in Table 6, and it is observed from the tabulated results that the proposed algorithm outperforms in terms of localization accuracy and residual energy.

## 5. Conclusions

A novel energy-efficient DV-Hop localization algorithm is implemented and simulated for WSNs. In this paper, the one-hop neighbors of beacon nodes are identified by broadcasting the tone request and reply mechanism over MAC to reduce the chance of collision. Further, the set of neighbor nodes are divided into two parts and the beacon node is selected as a common node in between the source and destination nodes which reduces energy consumption during transmission. The localization errors occurring at the second step of DV-Hop are reduced with refined hop-size distances of beacon nodes and localized unknown nodes are upgraded into helper nodes for assistance. To evaluate the performance of the proposed algorithm with the existing one, results are simulated in MATLAB. The simulated results demonstrated that the proposed algorithm performs 74.14%, 57.443%, 66.03%, and 34.36%, better as compared to [34,45,46,53] in terms of accuracy respectively.

## Figures and Tables

**Table 1:** Vicinity table of node B1 after node 1 reply.

Node Id	Location of the Neighbor Node	Indirect Transmission Cost	Common Node Id	Direct Transmission Cost	Status
1	_	0.3 J	_	0.3 J	0

**Table 2:** Vicinity table of node B1 after all neighbor nodes reply.

Node Id	Location of the Neighbor Node	Indirect Transmission Cost	Common Node	Direct Transmission Cost	Status
1	_	0.73 J	_	0.73 J	0
2	_	0.54 J	_	0.54 J	0
3	_	0.9 J	_	0.9 J	0
4	_	0.82 J	_	0.82 J	0
5	_	0.91 J	_	0.91 J	0
B3	(51, 42)	0.41 J	_	0.41 J	0
B4	(35, 82)	0.24 J	_	0.24 J	0

**Table 3:** Vicinity table of node B1 after finding $Com^{BN}$.

Node Id	Location of the Neighbor Node	Indirect Transmission Cost	Common Node	Direct Transmission Cost	Status
1	_	0.73 J	_	0.73 J	0
2	_	0.54 J	_	0.54 J	0
3	_	0.8 J	B3	0.9 J	1
4	_	0.75 J	B4	0.82 J	1
5	_	0.91 J	_	0.91 J	0
B3	(51, 42)	0.51 J	_	0.51 J	0
B4	(35, 82)	0.24 J	_	0.24 J	0

**Table 4:** Simulation parameters. Media access control (MAC).

Simulation Parameters	Value	Simulation Parameters	Value
Border length	100 × $100\;m^{2}$ to 500 × $500\;m^{2}$	MAC protocol	802.11 b
Total nodes	100–200	Initial energy	5 J
Beacon nodes	10 to 40%	Size of packets	512 bytes
Transmission range R	15–45 m	Maximum iterations	200
Network topology	Random	Network connectivity	2–15
DOI	0–0.3	Simulation time	400 s

**Table 5:** Minimum, maximum and average localization error (ALE) comparison of algorithms (DOI = 0). DV-Hop, evolutionary DV-Hop (EDV-Hop), improved DV-Hop (IDV-Hop), and advanced DV-Hop with teaching learning based optimization (ADV-Hop TLBO).

Algorithm	Maximum Error	Minimum Error	ALE
Basic DV-Hop [34]	35.144	9.224	19.47
EDV-Hop [45]	24.971	4.623	11.25
IDV-Hop [46]	30.312	7.0014	14.04
ADV-Hop TLBO [53]	16.308	0.735	7.3104
Proposed algorithm	10.304	0.1341	4.45

**Table 6:** Comparison of different performance evaluations.

Algorithm	Performance Evaluation						
	Localization Accuracy (%)	Average Residual Energy (%)	Transmission Range	MAC Incorporation	Network Type	An-isotropic Network	Packet Broadcasting
Basic DV-Hop [34]	80.53	82.56	Fixed	No	Homogenous	No	Whole network
IDV-Hop [46]	85.96	79.62	Fixed	No	Homogenous	No	Whole network
ADV-Hop TLBO [53]	92.68	76.34	Fixed	No	Homogenous	Yes	Whole network
EDV-Hop [45]	88.75	78.37	Fixed	No	Homogenous	No	Whole network
Proposed Algorithm	95.55	85.75	Variable	Yes	Heterogeneous	Yes	Within one-hop
